# Conducting a surveillance problem analysis on poor feedback from Reference Laboratory, Liberia, February 2016

**DOI:** 10.11604/pamj.supp.2017.27.1.12569

**Published:** 2017-05-28

**Authors:** Joseph Asamoah Frimpong, Maame Pokuah Amo-Addae, Peter Adebayo Adewuyi, Casey Daniel Hall, Meeyoung Mattie Park, Thomas Knue Nagbe

**Affiliations:** 1 Liberia Field Epidemiology Training Program, Monrovia, Liberia; 2 Rollins School of Public Health, Emory University, Atlanta, USA; 3 Ministry of Health, Monrovia, Liberia

**Keywords:** Public health, epidemiology, surveillance, problem analysis, laboratory surveillance

## Abstract

The laboratory plays a major role in surveillance, including confirming the start and end of an outbreak. Knowing the causative agent for an outbreak informs the development of response strategies and management plans for a public health event. However, issues and challenges may arise that limit the effectiveness or efficiency of laboratories in surveillance. This case study applies a systematic approach to analyse gaps in laboratory surveillance, thereby improving the ability to mitigate these gaps. Although this case study concentrates on factors resulting in poor feedback from the laboratory, practise of this general approach to problem analysis will confer skills required in analysing most public health issues. This case study was developed based on a report submitted by the district surveillance officer in Grand Bassa County, Liberia, as a resident of the Liberian Frontline Field Epidemiology Training Program in 2016. This case study will serve as a training tool to reinforce lectures on surveillance problem analysis using the fishbone approach. It is designed for public health training in a classroom setting and can be completed within 2 hours 30 minutes.

## How to use this case study

**General instructions:** a class of up to 20 trainees is ideal for a training sessions using this case study. The instructor facilitating the session should direct a participant to read a paragraph out loud, going around the room to give each participant a chance to read. Based on the type of question, the instructor may decide to divide the class into small groups for exercises, randomly identify a trainee to respond to the question, or engage the class in a group discussion of the answer. The aim of the interaction is to allow participants to learn from each other and not just from the instructor. Specific instructor’s notes are included with each question in the instructor’s version of this case study.

**Audience:** residents in Frontline Field Epidemiology Training Programs (FETP-Frontline), Field Epidemiology and Laboratory Training Programs (FELTPs), and others who are interested in this topic.

**Prerequisites:** for this case study, trainees should have received lectures on data quality, data quality auditing, and SWOT analysis.

**Materials needed:** flipchart or white board with markers

**Level of training and associated public health activity:** Novice – Data Quality Auditing

**Time required:** approximately 2-3 hours

**Language:** English

## Case study material

- Download the case study student guide (PDF - 1.50 MB)
- Request the case study facilitator guide
